# Glucose-sensitive hypothalamic nuclei traced through functional magnetic resonance imaging

**DOI:** 10.3389/fnins.2023.1297197

**Published:** 2023-12-11

**Authors:** Jorge Manuel, Eva Halbe, Ann Charlotte Ewald, Alex Hoff, Jens Jordan, Jens Tank, Karsten Heusser, Darius A. Gerlach

**Affiliations:** ^1^Institute of Aerospace Medicine, German Aerospace Center (DLR), Cologne, Germany; ^2^Institute for Neuroradiology, Hannover Medical School, Hanover, Germany; ^3^Department of Psychiatry and Psychotherapy, University of Bonn, Bonn, Germany; ^4^Medical Faculty, University of Cologne, Cologne, Germany

**Keywords:** fMRI, hypothalamus, energy metabolism, glucose regulation, functional connectivity, glucose-sensitive neural circuits, oral glucose tolerance test

## Abstract

**Introduction:**

Hypothalamic glucose-sensitive neural circuits, which regulate energy metabolism and can contribute to diseases such as obesity and type 2 diabetes, have been difficult to study in humans. We developed an approach to assess hypothalamic functional connectivity changes during glucose loading using functional magnetic resonance imaging (fMRI).

**Methods:**

To do so, we conducted oral glucose tolerance tests while acquiring functional images before, and 10 and 45 min after glucose ingestion in a healthy male and cross-sectionally in 20 healthy participants on two different diets.

**Results:**

At group level, 39 fMRI sessions were not sufficient to detect glucose-mediated connectivity changes. However, 10 repeated sessions in a single subject revealed significant intrinsic functional connectivity increases 45 min after glucose intake in the arcuate, paraventricular, and dorsomedial nuclei, as well as in the posterior hypothalamic area, median eminence, and mammillary bodies.

**Discussion:**

Our methodology allowed to outline glucose-sensitive hypothalamic pathways in a single human being and holds promise in delineating individual pathophysiology mechanisms in patients with dysglycemia.

## 1 Introduction

Glucose is sensed not only in the periphery like the tongue, pancreas, intestine, carotid bodies, or the portal vein; but also by glucose sensing neurons within the central nervous system. These neurons are mainly located in the hypothalamus and in the brainstem, and can be either excited or inhibited by glucose. In the brain, the most important region for sensing and integrating metabolic information is the hypothalamic arcuate nucleus which responds to glucose, insulin, leptin, ghrelin, as well as free fatty acids (Timper and Brüning, 2017). Neurons in the arcuate nucleus are connected to other hypothalamic regions like the paraventricular, dorsomedial, and ventromedial nuclei, together with the lateral hypothalamic area. These regions regulate energy homeostasis through the control of food intake and through autonomic nervous system efferents regulating pancreatic and hepatic function as well es energy expenditure (Timper and Brüning, 2017).

While the advent of new technologies such as optogenetics or chemogenetics resulted in rapid advances in knowledge of hypothalamic metabolic control in animals (Huang et al., 2022), the hypothalamus has been a difficult region to study in humans due to its small size, location deep within the brain, and its proximity to the ventricles and air-filled sinus. Hence, direct knowledge on neurally mediated glucose regulation in humans is scarce.

Previous functional magnetic resonance imaging (fMRI) studies on human hypothalamic glucose regulation often relied on region-of-interest based analyses in groups of subjects (Matsuda et al., 1999; Liu et al., 2000; Smeets et al., 2005b; van Opstal et al., 2015; Osada et al., 2017; Simon et al., 2020). However, these methodologies still have important limitations. First, inter-individual variability in glucose control, hypothalamic function, and anatomy introduce background noise, thus, reducing statistical power. Second, individual hypothalamic control mechanisms cannot be discerned, which is particularly relevant when studying patients with rare pathologies affecting these circuits. Third, region-of-interest based analyses are prone to selection bias of specific areas. Recent improvements in acquisition, processing, and analysis now permit to capture hypothalamic activity changes in groups of people without the need of subjectively defined regions, as shown by studies on pain (Schulte et al., 2017) or on autonomic reflexes (Napadow et al., 2007; Macefield and Henderson, 2016). We combined these advances with a glucose challenge in the scanner to elucidate hypothalamic glucose-sensitive pathways, both in the classical cross-sectional approach in multiple subjects, as well as using repeated measurements to visualize the pathways in a single subject.

## 2 Methods

The study was conducted at the German Aerospace Center in Cologne, Germany, in compliance with the Declaration of Helsinki (World Medical Association, 2013), and was approved by the ethics committee of the North Rhine medical board. The results in this paper correspond to an exploratory endpoint from a larger trial registered at the German Clinical Trials Register (DRKS00020750), whose primary results have been already published (De Gioannis et al., 2022). All participants provided informed consent.

We followed two approaches to study glucose-sensitive hypothalamic regions: a group and a single subject approach.

### 2.1 Group design

**Figure 1:**
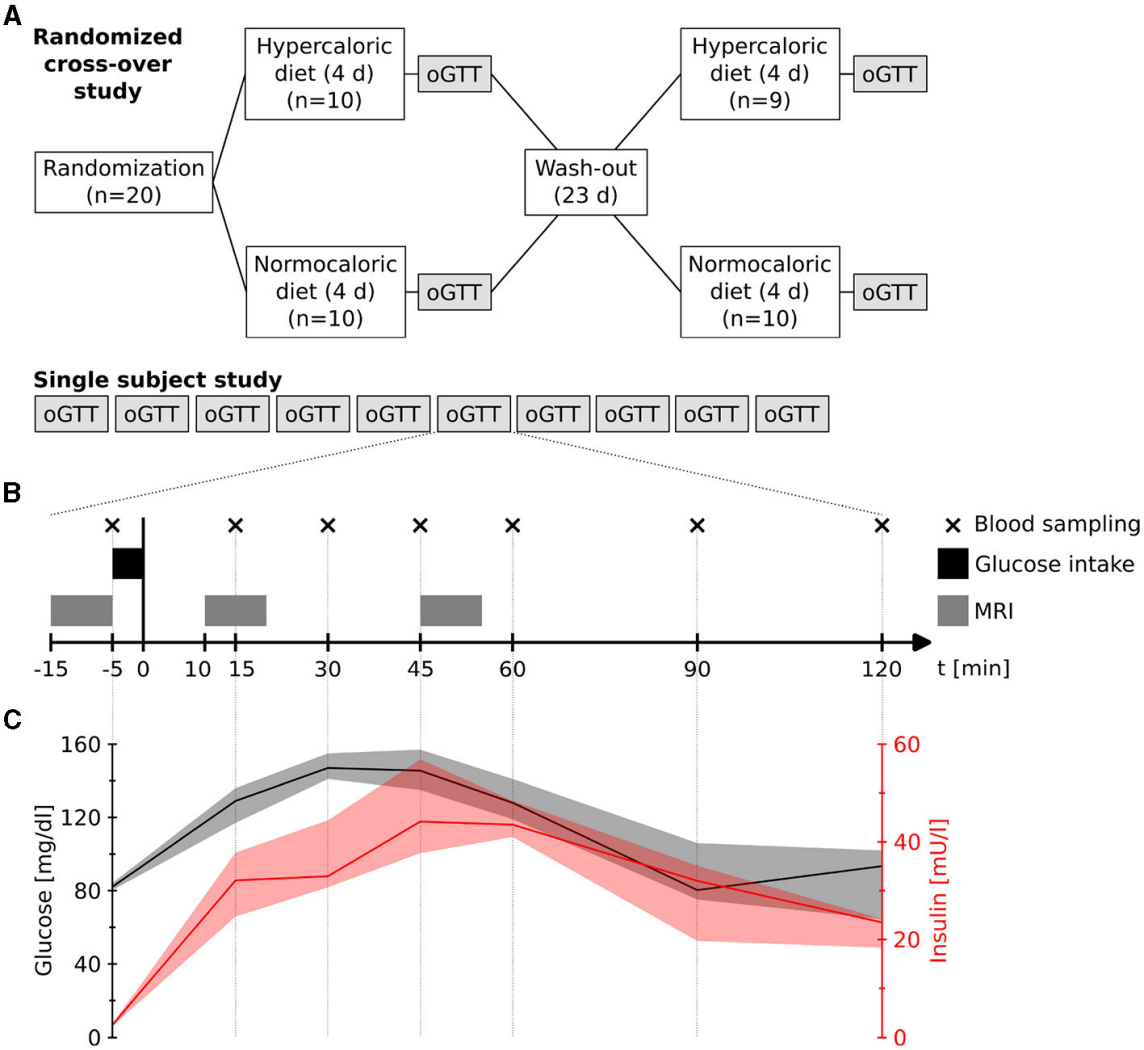
**(A)** Study designs. **(B)** Measurement procedure during each oral glucose tolerance test (oGTT) in both designs. Blood samples were taken at four days of the single subject study. **(C)** Measured plasma glucose and insulin levels (median and range) during four measurements of the single subject study.

We studied 20 healthy participants (7 women, 27 ± 8 years, 22.6 ± 2.0 kg/m^2^) who had been randomly assigned to two dietary interventions in a crossover design: four days normocaloric and four days modestly hypercaloric nutrition. Between the dietary blocks there was a washout phase of 23 days (Figure 1A). This washout phase ensured that the scans were 28 days apart (23 days washout + 4 days diet in between). Therefore, female participants were roughly in the same hormonal status. The normocaloric diet was adapted to the individual energy requirements by measuring resting metabolic rate through indirect calorimetry (Quark RMR, COSMED) and with the Freiburger questionnaire (Frey et al., 1999). The normocaloric diet supplied 9, 924 ± 1, 837 kJ of energy (84 ± 13 g protein, 74 ± 13 g fat, 310 ± 65 g carbohydrates, and 35 ± 5 g fiber). The hypercaloric diet provided 25 % more energy by increasing fat content to 132 ± 25 g. Following each dietary block, we conducted an oral glucose tolerance test on empty stomach with a pre-manufactured drink containing 75 g of glucose in 300 ml water (Dextrose O.G.-T., Roche). The glucose tolerance tests were carried out either at around 8:00 AM or 10:00 AM; always at the same time for each subject. We acquired functional images before, and 10 and 45 minutes after glucose ingestion. Each functional scan had a duration of 9.3 minutes (Figure 1B).

The subjects were recruited after obtaining a detailed anamnesis, physical examination, 12-lead electrocardiogram, blood pressure, and routine blood analysis. Eligible were healthy people aged 18–40 years (18–25 kg/m^2^, >55 beats per minute at rest) without recent body mass changes >3 kg, history of syncope, cardiac arrhythmia, smoking history, nor alcohol or drug abuse.

A detailed description of the procedures can be found in our previous publication (De Gioannis et al., 2022).

### 2.2 Single subject design

In an n-of-1 trial, we repeated ten oral glucose tolerance tests at different days on the same subject (healthy male, 56 years, 64 kg, 1.77 m) (Figure 1A). The glucose challenges were conducted at around 9:00 AM on empty stomach with the same drink as in the group design while acquiring functional scans at the same time points. Additionally, on four of the days, we measured venous plasma glucose and insulin levels at seven time points (-5, 15, 30, 45, 60, 90, and 120 min after glucose intake) (Figure 1B).

### 2.3 MRI acquisition and preprocessing

MRI acquisition and preprocessing were carried out as described previously (Manuel et al., 2020). In this study we used a 3 T Siemens Biograph mMR with a 32-channel head coil; the functional sequence had an isotropic resolution of 2 mm (T_R_ = 1.18s; T_E_ = 32ms; simultaneous multi-slice factor 6; partial Fourier 7/8); and the structural T_1_ had an isotropic resolution of 1 mm. We also acquired a reference scan for motion correction and template formation (equivalent to the functional sequence without through-plane acceleration), and reference scans for unwarping (two spin-echo images with opposed phase-encoding directions and without through-plane acceleration). All other parameters, especially all preprocessing steps, remained unaltered. Hence, we corrected motion and distortion [MCFLIRT (Jenkinson et al., 2002) and topup (Andersson et al., 2003)], extracted the brain [BET; Smith (2002)], applied grand mean scaling and a high pass filter (0.01 Hz), and normalized the data to a study template [ANTs; Avants et al. (2008)].

### 2.4 Data analysis

**Figure 2:**
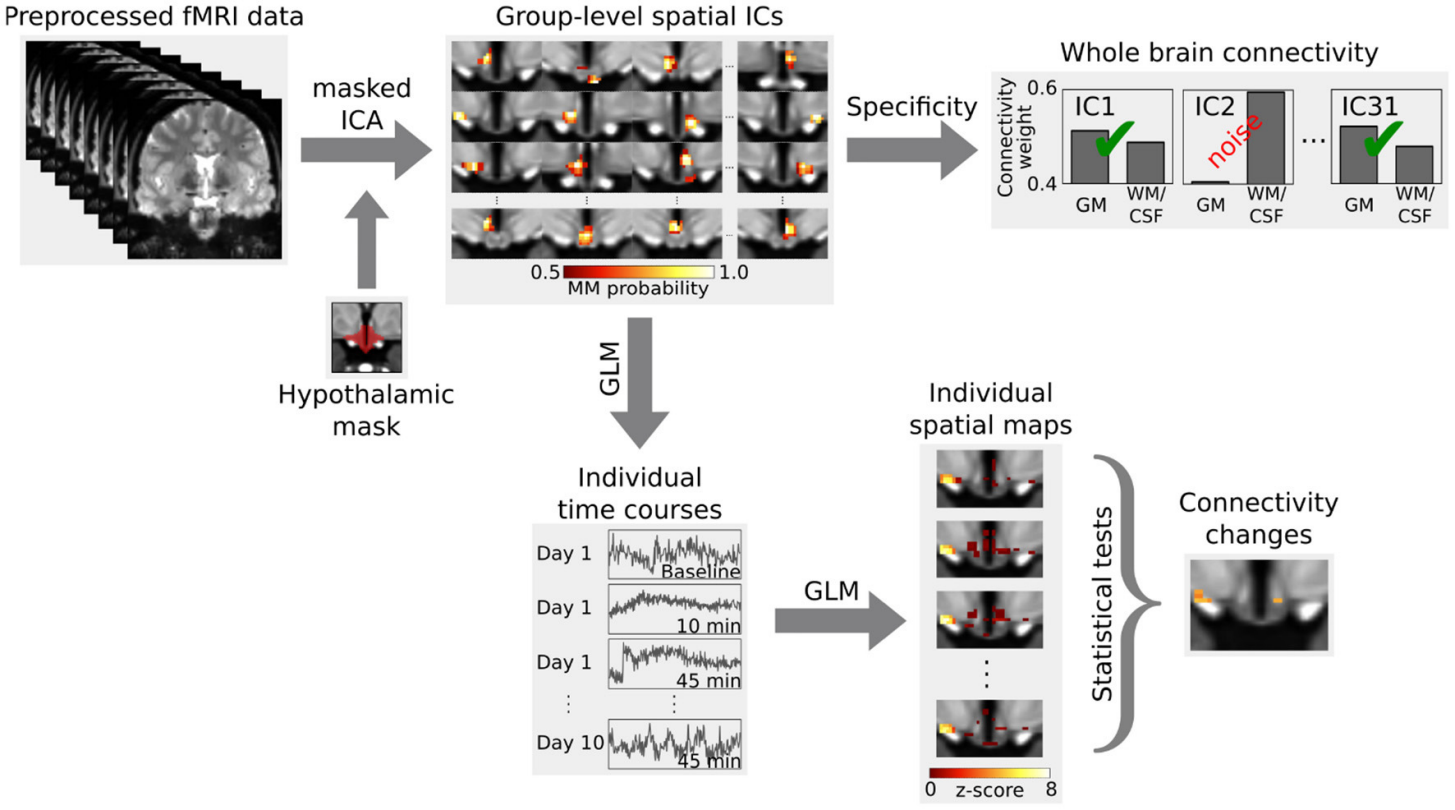
Analysis outline. Independent components (ICs) in the hypothalamus, derived from a masked independent component analysis (ICA), were tested for specificity by calculating their functional connectivity to the whole brain. Specific components were then tested for functional connectivity changes during the glucose challenge using a dual regression. The first step of the dual regression extracts the individual time courses of the BOLD signal for the group-level spatial ICs using a general linear model (GLM). These individual time courses are then used in a second step to obtain the individual spatial maps, which are then compared using inference tests. Figure adapted from Manuel et al. (2020).

For the analysis (Figure 2), we first performed a masked independent component analysis [mICA; Beissner et al. (2014)] on the concatenated functional data using a hypothalamic mask. ICA dimensionalities (45 for the group and 31 for the single subject data) were derived by maximizing reproducibility in a test-retest analysis between 1 and 100 dimensions using 20 random split-half samplings and Hungarian sorting of their cross-correlation matrix (mICA toolbox Moher Alsady et al., 2016). The specificity of independent components was tested by running a dual regression (Beckmann et al., 2009) to the whole brain. To do so, we calculated the weighted quotient of activation in gray matter versus white matter together with cerebrospinal fluid (probabilistic masks obtained using FAST Zhang et al., 2001). Two components in the group data and three components in the single subject data were considered unspecific for having a quotient smaller than one, i.e., they showed more functional connectivity to white matter and cerebrospinal fluid than to gray matter.

**Figure 3:**
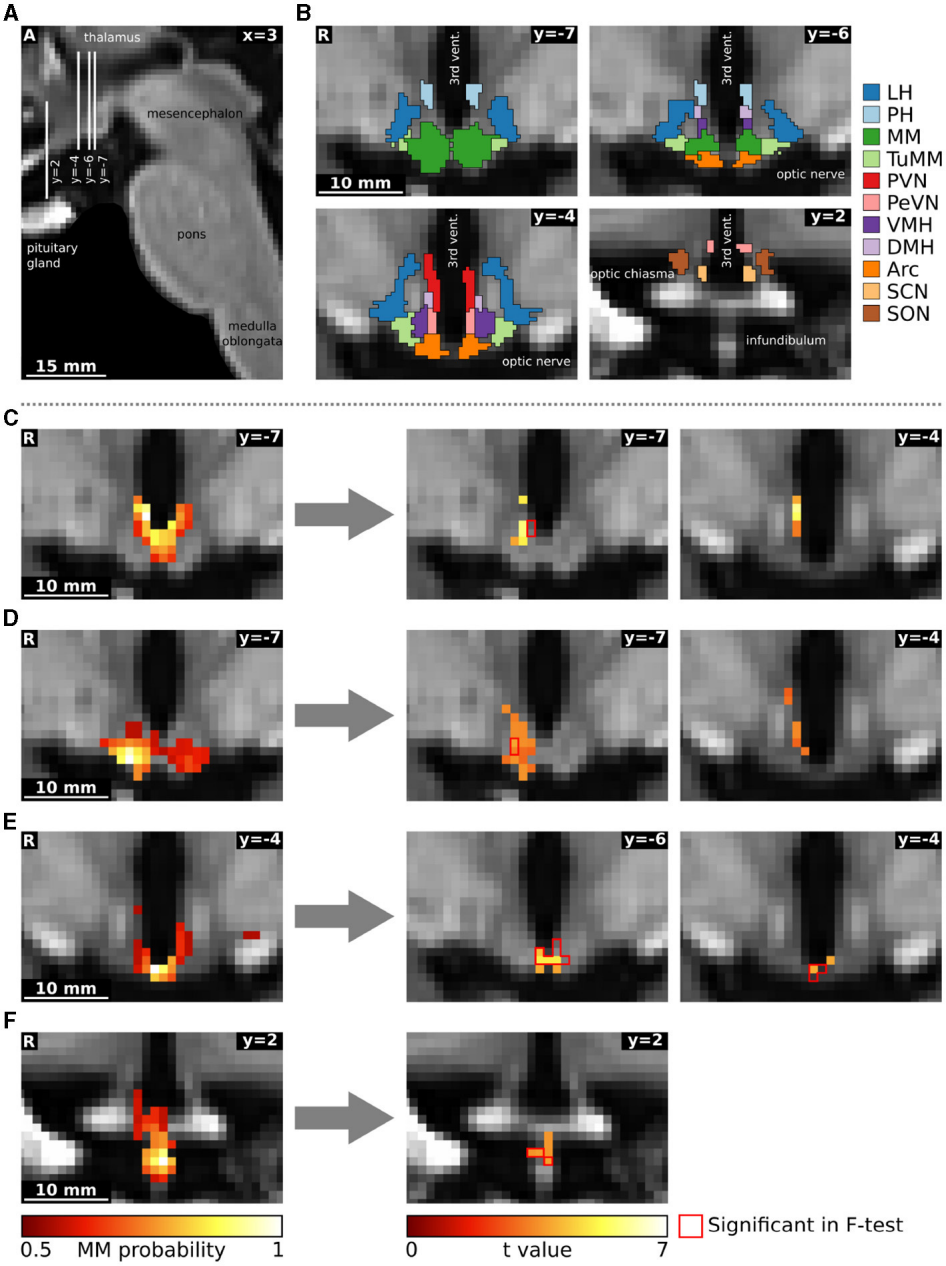
Functional connectivity changes following oral glucose ingestion in a single subject. **(A)** Sagittal image showing slice localization. **(B)** Hypothalamic regions from the atlas of Neudorfer et al. (2020). **(C–F)** Four independent components are depicted on the left as mixture model thresholded probability maps. On the right, we show functional connectivity changes obtained through a dual regression analysis. The red contour delineates statistically significant voxels in the F-test comparing all three conditions (rest, 10 min and 45 min after glucose intake), while the heat map describes overlapping functional connectivity increases 45 min after glucose intake. Although the omnibus and *post-hoc* results seem not to overlap in some slices, they do overlap in 3D. The reasons for the divergence of individual voxels are several and not trivial: (1) the number of parallel comparisons is different at omnibus (28) and at *post-hoc* (6) level leading to larger clusters after *p*-value correction; (2) non-parametric testing does not yield the exact same *p*-values when rerun; (3) omnibus F-tests with *post-hoc t*-tests are not completely equivalent (Chen et al., 2018); (4) we use threshold-free cluster enhancement (Smith and Nichols, 2009) which favours larger clusters. All images are in the study template transformed to MNI standard space. Arc, arcuate nucleus; DMH, dorsomedial hypothalamic nucleus; LH, lateral hypothalamic area; MM, mamillary bodies; PeVN, periventricular hypothalamic nucleus; PH, posterior hypothalamic area; PVN, paraventricular hypothalamic nucleus; SCN, suprachiasmatic nucleus; SON, supraoptic nucleus; TuMM, tuberomamillary nucleus; vent., ventricle; VMH, ventromedial hypothalamic nucleus.

Afterwards, we carried out a functional connectivity analysis using a dual regression to the hypothalamus. For the group data, we computed a 2 × 3 analysis of variance using multi-level block permutation [PALM, Winkler et al. (2015)]. Differences between the three time points in the single subject design were calculated with a non-parametric F-test followed by *post-hoc* paired two-sided *t*-tests [Randomise, Winkler et al. (2014)]. The results were thresholded at *p* < 0.05 using family-wise error correction with threshold-free cluster enhancement (Smith and Nichols, 2009). Moreover, we applied a Bonferroni correction to account for the multiple comparisons due to the multiple independent components (43 in the group and 28 in the single subject data). We identified hypothalamic regions with the help of a recently published MRI-atlas (Figure 3B) (Neudorfer et al., 2020). Additionally, we performed a supplementary hierarchical network clustering of the 28 single subject independent components based on the full cross-correlation matrix of the time series [FSLNets; Smith et al. (2013)].

We assessed hypothalamic signal-to-noise ratio in the data in two different ways. First, we computed the temporal signal-to-noise ratio (tSNR) by dividing the mean by the standard deviation of the signal within the hypothalamic mask. Second, we calculated the contrast-to-noise ratio (CNR) as the ratio between the standard deviation of the signal in the third block (45 min after glucose ingestion) and baseline's standard deviation (Welvaert and Rosseel, 2013).

## 3 Results

### 3.1 Group

We segmented the group data into 45 regions using a masked independent component analysis of the fMRI time series, followed by removal of two unspecific components. The remaining 43 independent components were subjected to a dual regression analysis combined with an analysis of variance (Figure 2). None of the components showed significant connectivity changes; neither between time points, nor between diets, nor any interactions. We did not find any statistical trends (*p* < 0.1) either.

### 3.2 Single subject

Venous plasma glucose and insulin levels followed the expected time courses after oral glucose in a healthy male (56 years, 64 kg, 1.77 m) (Jarrett et al., 1972). Plasma glucose rapidly increased and peaked 30 min after glucose intake (median: 147 mg/dl; range: 141–155 mg/dl), while insulin also increased with a maximum approximately 15 min thereafter (median: 44.2 mU/l; range: 37.7–56.9 mU/l) (Figure 1C).

The single subject fMRI-data was analyzed similarly to the group data. Three out of the 31 hypothalamic regions were deemed as noise. The remaining 28 independent components (Supplementary Table S1) were tested for their involvement in glucose regulation using a dual regression analysis (Figure 2). In a non-parametric F-test, four components showed statistically significant differences in functional connectivity between the three time points (baseline, 10 and 45 min after glucose intake). *Post-hoc* paired *t-*tests revealed that these connectivity changes were all between baseline and 45 min after glucose intake. By overlaying an anatomical atlas onto the images, we identified eight hypothalamic nuclei within these four regions: the arcuate, paraventricular, dorsomedial, periventricular, and tuberomamillary nuclei, mamillary bodies, median eminence, as well as the posterior hypothalamic area. Moreover, two regions encompassing the mamillary bodies increased their functional connectivity to the ventromedial hypothalamic nucleus (see Figure 3, as well as Supplementary Tables S1, S2).

Specifically, the first component, encompassing the mamillary bodies, posterior hypothalamic area, and the paraventricular and dorsomedial hypothalamic nuclei, increased its intrinsic functional connectivity, as well as its connectivity to the nearby ventromedial hypothalamic nucleus (Figure 3C). The second independent component, which included the mamillary bodies, and the arcuate and tuberomamillary nuclei, also increased its intrinsic functional connectivity, as well as its connectivity to the dorsomedial, ventromedial, and periventricular hypothalamic nuclei (Figure 3D). Both the third (mamillary bodies, arcuate and periventricular nuclei) and fourth (median eminence) regions increased their intrinsic connectivity 45 min after glucose ingestion compared to baseline (Figures 3E, F).

A hierarchical network clustering of the 28 independent components (Supplementary Figure S1) showed that the first three components, all of which partially encompass the mamillary bodies, are closer to each other than to the component located in the median eminence. We did not find any statistically significant difference in the network across the three time points.

### 3.3 Signal-to-noise ratio

**Figure 4:**
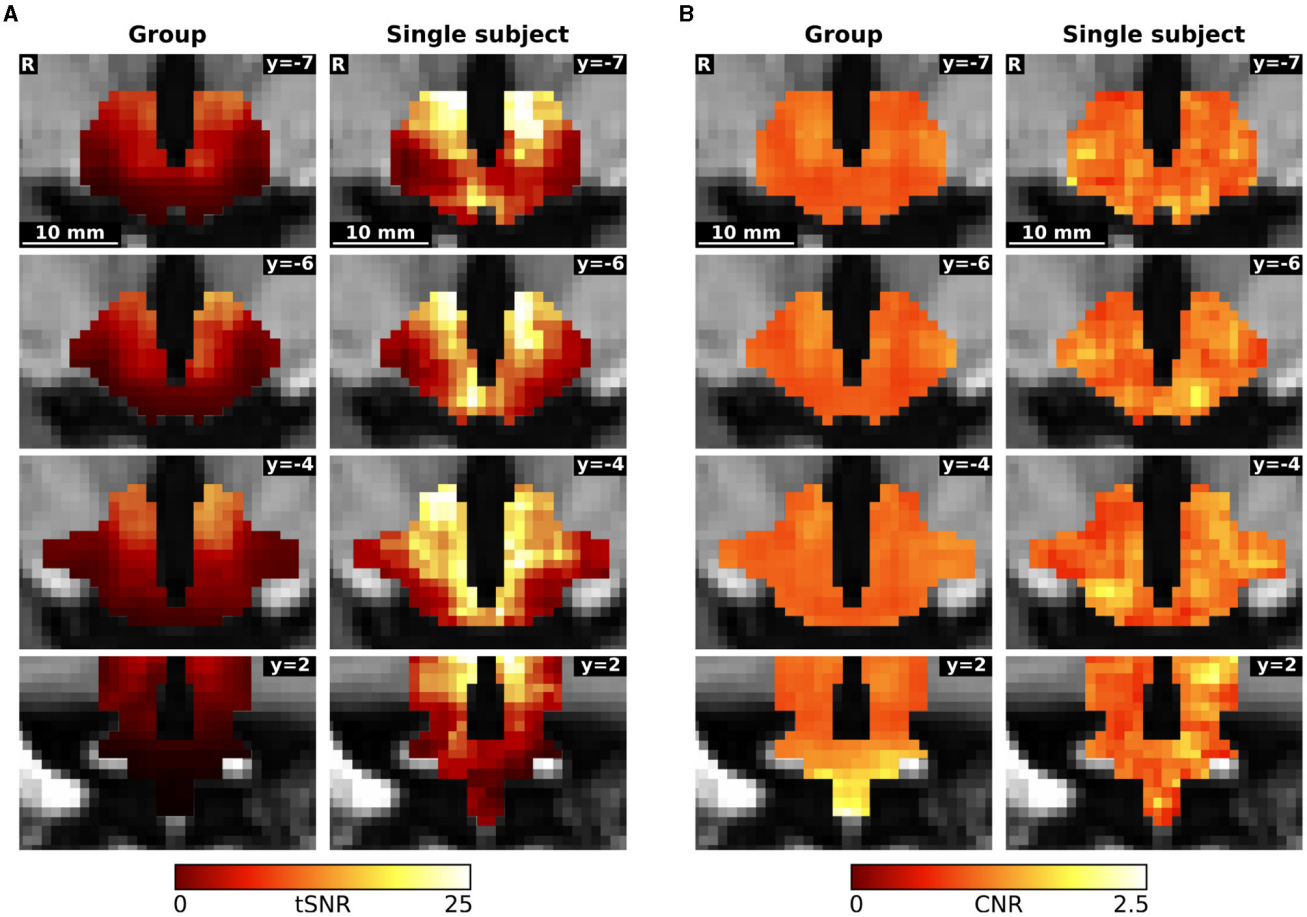
Comparison of **(A)** temporal signal-to-noise ratio (tSNR) and **(B)** contrast-to-noise ratio (CNR) between the concatenated group and single subject data.

The overall hypothalamic temporal signal-to-noise ratio (tSNR) of the concatenated group data was 8.97, whereas the tSNR of the concatenated data of the n-of-1 trial was 15.72. Contrast-to-noise ratio (CNR) in the hypothalamus was similar in the group and in the single subject approach (1.09 vs. 1.10 respectively). The single subject CNR appears to be less smoothed, having more structure (Figure 4).

These values for the concatenated data contrast with the values for the individual measurement blocks. The tSNR was significantly higher in the group than in the single subject trial (39.80 ± 4.32 vs. 30.61 ± 2.06; *p* < 0.001), while the contrast-to-noise ratio was lower in the group data (1.04 ± 0.09 vs. 1.14 ± 0.11; *p* = 0.0428).

## 4 Discussion

Previous fMRI studies investigating glucose regulation in humans (see Supplementary Table S3) have shown BOLD (blood oxygenation level dependent) signal decreases in the lower posterior and upper anterior hypothalamus following glucose ingestion (Matsuda et al., 1999; Liu et al., 2000; Flanagan et al., 2012). A recent study found signal decreases in the ventromedial hypothalamic and in the arcuate nucleus, and signal increases in the lateral hypothalamic area after glucose ingestion (Osada et al., 2017). The hypothalamic signal decreases, which have been observed after glucose, but not after water, aspartame or maltodextrin ingestion (Smeets et al., 2005a), are dose dependent (Smeets et al., 2005b). Moreover, the BOLD-signal decrease lasts longer after glucose ingestion than after intravenous injection, similarly to plasma insulin levels (Smeets et al., 2007; Purnell et al., 2011; Simon et al., 2023). Intragastric glucose injection also leads to a signal decrease in the hypothalamus (Simon et al., 2020). The hypothalamic response to glucose is altered in obesity (Matsuda et al., 1999) and in type 2 Diabetes mellitus (Vidarsdottir et al., 2007; Teeuwisse et al., 2012). In patients with Anorexia nervosa, one study found no difference after oral glucose (van Opstal et al., 2015), whereas another found significant differences in hypothalamic signal intensity after intragastric glucose (Simon et al., 2020).

In contrast to all aforementioned studies, our study was not designed to compare absolute intensity changes as we acquired three time series instead of one, and BOLD-signal intensity is not preserved across series. However, our higher spatial and temporal resolution allowed us to use a data driven approach to segment the hypothalamus in functionally independent regions, and to calculate functional connectivity changes between the time points.

Our results show that hypothalamic glucose-sensitive regions can be delineated through fMRI in a single subject. In fact, while ten repeated fMRI studies were sufficient to delineate glucose-mediated hypothalamic connectivity changes in a single subject, a cross-sectional study in 20 subjects with 39 fMRI scans overall failed to show such a response. The group data also did not reveal statistically significant differences between diets. The finding is in line with our previous findings that four days moderate hypercaloric diet do neither alter orthostatic tolerance, nor blood pressure and heart rate regulation which are controlled at the level of the brainstem (De Gioannis et al., 2022).

**Figure 5:**
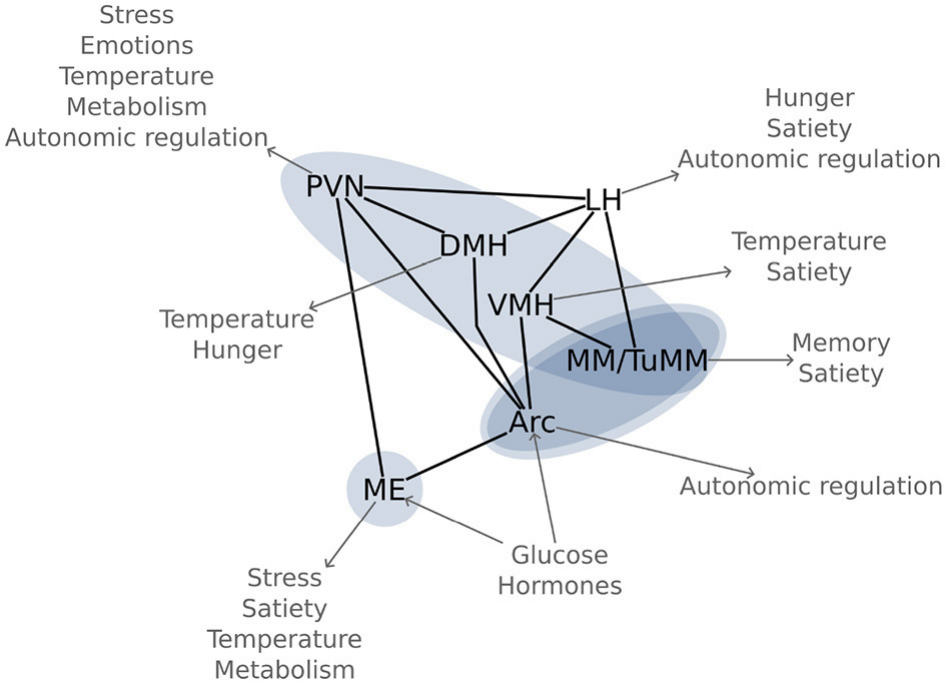
Glucose sensitive hypothalamic pathways: Most important hypothalamic nuclei for glucose regulation and their function in energy homeostasis. The blue ovals highlight the significant functional connectivity changes between the regions, as found in Figure 3. Arc, arcuate nucleus; DMH, dorsomedial hypothalamic nucleus; LH, lateral hypothalamic area; ME, median eminence; MM, mamillary bodies; PVN, paraventricular hypothalamic nucleus; TuMM, tuberomamillary nucleus; VMH, ventromedial hypothalamic nucleus.

Using an n-of-1 trial approach, we visualized six out of the seven most important hypothalamic regions for energy homeostasis (Figure 5). The arcuate nucleus is the main region for sensing and integrating metabolic information. Being capable of sensing leptin and ghrelin, as well as glucose and insulin (Korf and Møller, 2021); the arcuate nucleus is perfectly equipped for controlling energy homeostasis. This nucleus does so by regulating food intake, autonomic outflow, and pancreatic activity through its neural connections to other nuclei like the paraventricular, dorsomedial, and ventromedial hypothalamic nuclei, as well as to regions in the brainstem like the parabrachial, solitary, and dorsal vagal nuclei (Timper and Brüning, 2017). This network around the arcuate nucleus has been coined the melanocortin system due to the importance of melanocortin receptors.

Close to the arcuate nucleus, in the infundibular stalk, lies the median eminence, a neurohaemal region. The median eminence is not only important for releasing hormones into the hypophyseal portal system, but also as a sensory region. The floor of the third ventricle around the arcuate nucleus and the median eminence is equipped with specialized glia cells, the tanycytes, which are responsible for modulating the transport of hormones between systemic circulation and cerebrospinal fluid. The fact that an ablation of this region increases body fat content (Yoo et al., 2020), highlights its importance in energy homeostasis.

Another region that we observed and which is involved in metabolic regulation is the ventromedial hypothalamic nucleus. Located directly cranial to the arcuate nucleus, the ventromedial nucleus has long been known to lead to obesity if damaged (Hetherington and Ranson, 1942). There has been some controversy regarding its involvement in metabolic regulation due to its proximity to the arcuate nucleus; however, the current accepted notion is that the ventromedial hypothalamic nucleus regulates satiety (King, 2006).

Cranial to the ventromedial nucleus lies the dorsomedial hypothalamic nucleus, which is mainly responsible for thermoregulation and for circadian rhythms through connections to the suprachiasmatic nucleus (Bernardis and Bellinger, 1998). Moreover, this region is also involved in metabolic regulation, and, when damaged, leads to appetite loss (Bellinger and Bernardis, 2002). Indeed, the dorsomedial hypothalamic nucleus may be the main region for food-entrainable circadian rhythms, although this hypothesis is still debated (Moriya et al., 2009).

The paraventricular hypothalamic nucleus is a crucial integration hub, connecting via afferents and efferents to other nuclei in the hypothalamus, thalamus, brainstem, and the amygdala. Moreover, the paraventricular hypothalamic nucleus is directly involved in humoral responses through the neurohypophysis, as well as through the hypophyseal portal system via the median eminence (Coote, 2004). Through its connections, the paraventricular nucleus coordinates stress responses, emotions, circadian rhythms, body temperature, reproduction, and metabolism. Bilateral lesions of the paraventricular nucleus lead to obesity (Leibowitz et al., 1981).

Three of the independent components that we identified included the mamillary bodies. Although best known for their involvement in memory, the mamillary bodies also seem to be important for other processes since they have been involved in heart failure (Kumar et al., 2009) and obesity (Gold et al., 1972). The tuberomamillary nucleus, a key region of the histaminergic system, has also been involved in satiety (Sakata et al., 2003). Nonetheless, our knowledge on these aspects is still scarce and ought yet to be studied thoroughly.

To our knowledge, the posterior hypothalamic area is not directly involved in energy homeostasis. However, due to its connections to the brainstem (Coote, 2004), the posterior hypothalamic area may be involved in autonomic regulation. Moreover, this region has been associated with adaptive behavior because of its links to the hippocampus and amygdala (Abrahamson and Moore, 2001). A word of caution should be given here: we reported all the nuclei which had at least 5% of their area within the independent component. The posterior hypothalamic area fulfilled this criterion, but was located at the verge of the component, and could, thus, be an effect of our limited resolution.

In our experiment, we did not observe functional connectivity changes in the lateral hypothalamic area. This region contains both, glucose-excited and glucose-inhibited neurons, and its dysfunction may lead to obesity or to leanness depending on the affected neurons (Burdakov et al., 2013). The simultaneous excitation and inhibition by glucose in nearby regions can lead to a net zero change in the fMRI signal, which integrates the response of thousands of neurons in each voxel. Thus, glucose ingestion might not be the ideal stimulus for visualizing the lateral hypothalamic area using fMRI.

Most likely, we did not observe connectivity differences in the group data due to large inter-individual variability in the response to oral glucose. Although having almost four times as many data points and a larger temporal SNR in the separate measurements, the concatenated group data had only 57% of the single subject's tSNR within the hypothalamus. These numbers point to a large inter-individual variability. It is known that there are at least two different response types to glucose ingestion; the most common one being an initial increase in plasma glucose levels with a steady decrease afterwards towards baseline. However, also oscillating forms have been observed; i.e., rapid increases in plasma glucose followed by an overcompensation and a second increase in plasma glucose in the second hour (Tschritter et al., 2003). Other factors that could affect the response are circadian (Jarrett et al., 1972), age, and sex differences (Boyns et al., 1969). We did not characterize the individual glucose and insulin responses prior to the oral glucose tolerance tests. This problem should be addressed in future studies, as even relatively small delays of 5–10 min in the response could greatly affect the fMRI signal. One invasive solution to this problem would be to clamp plasma glucose levels (Heise et al., 2016). Finally, inter-individual differences in hypothalamic anatomy and regulation could introduce variability in the group results.

Our single subject fMRI approach combining data-driven parcellation with functional connectivity analysis during a glucose challenge delineated for the first time individual glucose-sensitive hypothalamic pathways in a human being. Indeed, the approach was more powerful for gaining insight into human hypothalamic physiology than studying multiple persons in a cross-sectional fashion. Glucose ingestion engaged six out of the seven most important hypothalamic regions for energy homeostasis. This study demonstrates that glucose-sensitive hypothalamic pathways can be depicted when comparing functional connectivity during peak insulinemia and baseline. Nonetheless, future studies should address the number of repetitions needed to robustly identify these regions, as well as validate the results in more subjects. Individual hypothalamic fMRI might help to elucidate human physiology and disease mechanisms in single patients. The approach could have particular relevance in studies tracing hypothalamic function in patients with rare disorders affecting this region of the brain.

## Data availability statement

The raw data supporting the conclusions of this article will be made available by the authors, without undue reservation.

## Ethics statement

The studies involving humans were approved by the Ethics Committee of the North Rhine medical board. The studies were conducted in accordance with the local legislation and institutional requirements. The participants provided their written informed consent to participate in this study.

## Author contributions

JM: Conceptualization, Data curation, Formal analysis, Methodology, Software, Visualization, Writing—original draft, Writing—review & editing. EH: Formal analysis, Investigation, Writing—review & editing. AE: Conceptualization, Writing—review & editing. AH: Investigation, Writing—review & editing. JJ: Conceptualization, Writing—review & editing. JT: Conceptualization, Writing—review & editing. KH: Conceptualization, Investigation, Writing—review & editing. DG: Conceptualization, Data curation, Formal analysis, Methodology, Supervision, Writing—review & editing.
