# Supplementary material for: Glucose-sensitive hypothalamic nuclei traced through functional magnetic resonance imaging
Source: Front Neurosci. 2023 Dec 11;17:1297197. doi: 10.3389/fnins.2023.1297197 (PMC10749345; doi:10.3389/fnins.2023.1297197)
Supplement: Supplementary file 1 [file Data_Sheet_1.PDF]

Supplementary Material

1 SUPPLEMENTARY TABLES AND FIGURES

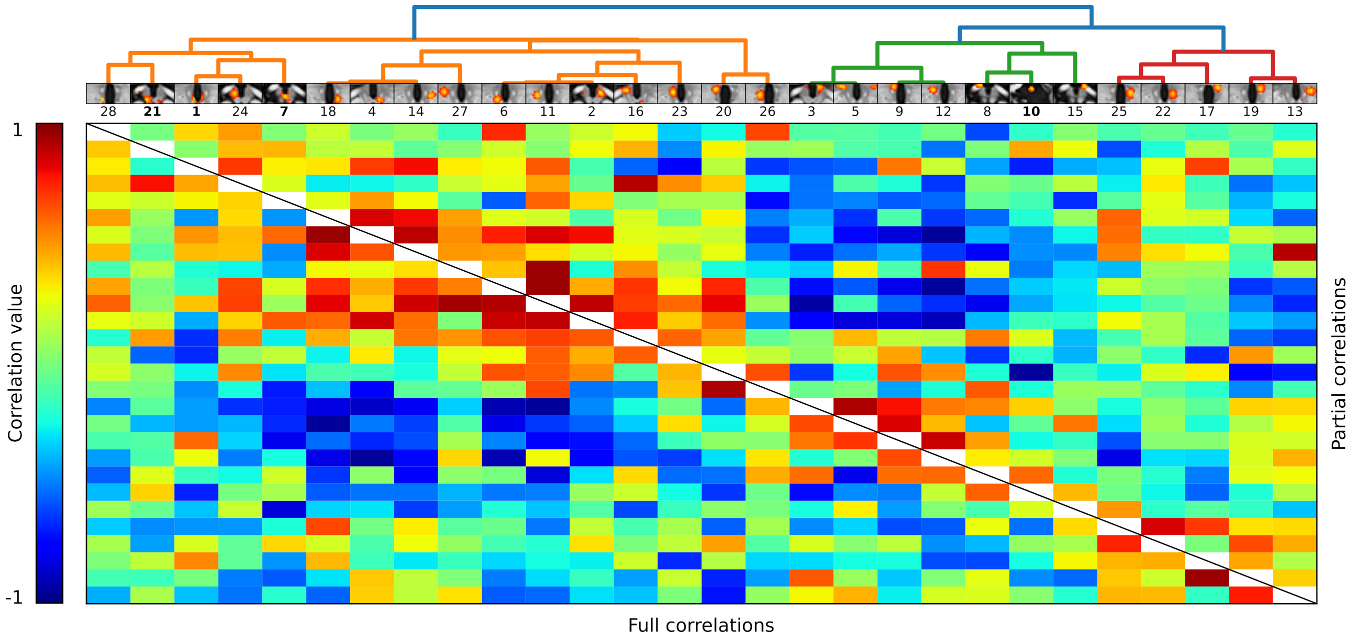

Figure S1. Hierarchical network clustering of the 28 independent components using the FSLNets package (Smith et al., 2013). The clustering is based on the full cross-correlation matrix of the time series from the 28 independent components. The partial correlation matrix is shown above the diagonal, whereas the full correlation matrix is depicted below the diagonal. The four independent components from figure 3 have been highlighted in bold.

Table S1: Coordinates and anatomical identification of spatial independent components. Note that the median eminence is not covered by the hypothalamic atlas of Neudorfer et al. (2020); we identified it by following anatomical descriptions (Weaver and Bucy, 1940).

	Maximum [mm]			Gravity centre [mm]			
IC	x	y	z	x	y	z	Nuclei
1	2	-7	-10	0.4	-7.1	-11.1	Mamillary bodies, posterior hyp. area, paraventricular hyp. ncl., dorsomedial hyp. ncl.
2	-5	-3	-15	-4.3	-2.8	-14.4	Ventromedial hyp. ncl., anterior hyp. area, tuberomamillary ncl., arcuate ncl.
3	-3	1	-13	-4.1	0.4	-12.7	Anterior hyp. area, lateral hyp. area, supraoptic ncl., ventromedial hyp. ncl., suprachiasmatic ncl.
4	-3	-7	-12	-3.4	-6.7	-12.9	Mamillary bodies, lateral hyp. area, tuberomamillary ncl., arcuate ncl.
5	9	1	-12	9.4	0.9	-11.6	Lateral hyp. area, supraoptic ncl.
6	5	-8	-10	4.6	-7.4	-10.3	Lateral hyp. area, mamillary bodies, posterior hyp. area
7	1	-5	-16	0.1	-4.7	-15.2	Arcuate ncl., mamillary bodies, periventricular hyp. ncl.
8	5	2	-16	5.6	2.1	-16.5	Supraoptic ncl., suprachiasmatic ncl.
9	4	0	-8	4.4	0.9	-9.1	Lateral hyp. area, paraventricular hyp. ncl., anterior hyp. area, medial preoptic ncl.
10	0	2	-23	0.6	1.4	-21.4	Median eminence
11	7	-6	-9	7.4	-4.9	-10.0	Lateral hyp. area, tuberomamillary ncl.
12	7	-2	-9	7.7	-1.2	-9.4	Lateral hyp. area
13	-9	-2	-11	-7.9	-2.1	-11.4	Lateral hyp. area, supraoptic ncl., anterior hyp. area
14	-9	-5	-11	-7.8	-5.1	-10.1	Lateral hyp. area, tuberomamillary ncl.
15	-5	1	-17	-3.9	1.2	-16.7	Supraoptic ncl., suprachiasmatic ncl.
16	6	-1	-13	6.8	-1.0	-13.2	Lateral hyp. area, anterior hyp. area, mamillary bodies, supraoptic ncl., ventromedial hyp. ncl.
17	-4	0	-5	-4.9	-0.5	-5.5	Lateral hyp. area
18	-5	-8	-8	-4.3	-7.4	-7.4	Lateral hyp. area, posterior hyp. area
19	-5	-1	-9	-4.4	-0.7	-8.4	Lateral hyp. area, paraventricular hyp. ncl., medial preoptic ncl.
20	6	-1	-6	5.3	-1.5	-5.5	Lateral hyp. area
21	4	-7	-15	2.9	-6.6	-14.1	Mamillary bodies, arcuate ncl., tuberomamillary ncl.
22	-5	-4	-10	-4.5	-4.0	-9.9	Lateral hyp. area, anterior hyp. area, ventromedial hyp. ncl., dorsomedial hyp. ncl.
23	5	-2	-10	4.7	-2.9	-10.7	Anterior hyp. area, lateral hyp. area, ventromedial hyp. ncl., dorsomedial hyp. ncl., paraventricular hyp. ncl., tuberomamillary ncl., periventricular hyp. ncl.
24	4	-3	-15	4.2	-2.4	-15.1	Arcuate ncl., ventromedial hyp. ncl., periventricular hyp. ncl., anterior hyp. area, tuberomamillary ncl.
25	-5	-3	-6	-5.1	-3.8	-6.4	Lateral hyp. area
26	5	-5	-7	5.2	-5.1	-6.3	Lateral hyp. area, posterior hyp. area

27	10	-3	-11	11.0	-2.7	-10.3	Lateral hyp. area
28	4	-8	-7	4.2	-7.9	-6.9	Posterior hyp. area, lateral hyp. area, anterior hyp. area, supraoptic ncl.

Table S2: Coordinates and anatomical identification of dual regression clusters of figure 3. Note that the median eminence is not covered by the hypothalamic atlas of Neudorfer et al. (2020); we identified it by following anatomical descriptions (Weaver and Bucy, 1940).

		Max.	Size	Maximum [mm]			Gravity centre [mm]			
Fig 3	IC	t-value	[mm^3]	x	y	z	x	y	z	Nuclei
c	1	5.97	36	3	-5	-10	3.0	-5.7	-10.7	Mamillary bodies, paraventricular hyp. ncl., dorsomedial hyp. ncl., ventromedial hyp. ncl., posterior hyp. area
d	21	4.85	61	2	-5	-13	3.4	-6.1	-12.4	Mamillary bodies, dorsomedial hyp. ncl., periventricular hyp. ncl., fornix, ventromedial hyp. ncl.
e	7	5.19	15	-1	-5	-14	-0.2	-5.7	-15.1	Arcuate ncl., mamillary bodies
f	10	4.36	14	0	1	-21	0.7	1.2	-21.1	Median eminence

Table S3: fMRI studies investigating glucose regulation.

**Matsuda et al. (1999)**	
Paradigm	Oral glucose and water
Subjects	10 healthy (5 men), 10 obese (5 men)
fMRI parameters	Mid-sagittal slice (1x1x10 mm^3). Measured till 40 min after stimulus.
Results	BOLD-signal decrease in lower posterior and upper anterior hypothalamus. Less and delayed signal drop in obese subjects. Response correlated with fasting plasma glucose and insulin.
Comments	
**Liu et al. (2000)**	
Paradigm	Oral glucose and water
Subjects	21 healthy (11 men)
fMRI parameters	Mid-sagittal slice (1x1x10 mm^3). Measured till 38 min after stimulus.
Results	BOLD-signal decrease in hypothalamus, similar to Matsuda et al. (1999).
Comments	
**Smeets et al. (2005a)**	
Paradigm	Oral glucose, water, aspartame, and maltodextrin
Subjects	5 healthy men
fMRI parameters	Mid-sagittal slice (1.1x0.8x10 mm^3). Measured till 30 min after stimulus.
Results	BOLD-signal decrease only seen after glucose ingestion.
Comments	No fMRI images shown
**Smeets et al. (2005b)**	
Paradigm	Oral glucose (two doses) and water
Subjects	15 healthy men
fMRI parameters	Mid-sagittal slice (1.1x0.8x10 mm^3). Measured till 30 min after stimulus.
Results	BOLD-signal decrease after glucose ingestion. Dose dependent in the upper anterior hypothalamus.
Comments	No fMRI images shown
**Smeets et al. (2007)**	
Paradigm	Oral glucose, oral water, intravenous glucose, and intravenous saline
Subjects	7 healthy men
fMRI parameters	Mid-sagittal slice (0.8x0.9x12 mm^3). Measured till 30 min after stimulus.
Results	Intravenous glucose results in a transient signal decrease in hypothalamus (till around 15 min) compared to the prolonged decrease after oral glucose. Plasma insulin peaked at 15 min after intravenous glucose, whereas it continued to increase at least till 60 min after oral glucose.
Comments	No fMRI images shown
**Vidarsdottir et al. (2007)**	
Paradigm	Oral glucose and water
Subjects	10 healthy men and 7 men with type 2 Diabetes mellitus
fMRI parameters	Mid-sagittal slice (0.8x0.9x12 mm^3). Measured till 30 min after stimulus.
Results	No hypothalamic BOLD-signal decrease in Diabetes mellitus after glucose ingestion.
Comments	No fMRI images shown

Purnell et al. (2011)

Paradigm	Intravenous glucose, fructose, and saline
Subjects	9 healthy (3 men)
fMRI parameters	Two mid-sagittal slices (1x1x5 mm^3). Measured till 35 min after stimulus.
Results	No hypothalamic changes after glucose or fructose injection. Cortical signal decrease after fructose and increase after glucose injection. No regional differences in hypothalamus or in brain.
Comments	No fMRI images shown

Flanagan et al. (2012)

Paradigm	Oral glucose and water
Subjects	7 healthy (4 men)
fMRI parameters	Whole brain (2x2x2 mm^3). Measured till 35 min after stimulus.
Results	Less BOLD-signal increase in hypothalamus after glucose than after water (no detrending of BOLD time series).
Comments	No fMRI images shown. Only motion correction.

Teeuwisse et al. (2012)

Paradigm	Oral glucose. Normal diet vs. 4-day hypocaloric diet (450 kcal).
Subjects	10 men with type 2 Diabetes mellitus
fMRI parameters	Mid-sagittal slice (0.8x0.9x14 mm^3). Measured till 30 min after stimulus.
Results	Larger hypothalamic BOLD-signal decrease after hypocaloric diet. Insulin sensitivity improved after hypocaloric diet.
Comments	No fMRI images shown

van Opstal et al. (2015)

Paradigm	Oral glucose
Subjects	11 healthy women, 10 women with Anorexia nervosa
fMRI parameters	Mid-sagittal slice (0.8x0.8x14 mm^3). Measured till 30 min after stimulus.
Results	No differences between Anorexia nervosa and healthy women.
Comments	No fMRI images shown

Osada et al. (2017)

Paradigm	Oral glucose
Subjects	12 healthy (6 male)
fMRI parameters	Whole brain (1.25x1.25x1.25 mm^3). Measured till 44 min after stimulus.
Results	BOLD-signal decrease in the ventromedial hypothalamic and in the arcuate nucleus after glucose ingestion. Signal increase in the lateral hypothalamic area.
Comments	Parcellation of hypothalamus using functional connectivity of hypothalamic voxels to cortex seeds. Comprehensive preprocessing (slice timing, motion, bandpass, noise regression).

Al-Zubaidi et al. (2018)

Paradigm	Oral glucose. Fasting (36 h) vs. satiety.
Subjects	24 healthy men
fMRI parameters	Whole brain (3x3x3 mm^3). Measured till 30 min after stimulus.
Results	Left thalamus shows differences in BOLD-signal after glucose ingestion during fasting and satiety.
Comments	

**Al-Zubaidi et al. (2020)**	
Paradigm	Oral glucose. Hunger vs. satiety.
Subjects	20 (24-4) healthy men
fMRI parameters	Whole brain (3x3x3 mm^3). Measured till 30 min after stimulus.
Results	Hunger increased forward and decreased backward connectivity from posterior to anterior insula.
Comments	
**Simon et al. (2020)**	
Paradigm	Intragastric glucose and water
Subjects	28 healthy women, 24 women with Anorexia nervosa, 24 obese women
fMRI parameters	Whole brain (2x2x2 mm^3). Measured till 26 min after stimulus.
Results	BOLD-signal decrease in healthy women in the whole hypothalamus, whole amygdala, and whole Ncl. accumbens after glucose injection . No signal decrease in subjects with anorexia or obesity.
Comments	
**Simon et al. (2023)**	
Paradigm	Intravenous glucose and saline
Subjects	31 healthy (14 male)
fMRI parameters	Whole brain (2x2x2 mm^3). Measured till 24 min after stimulus.
Results	BOLD-signal decrease in whole hypothalamus after glucose injection. Decreased functional connectivity between hypothalamus and medial prefrontal cortex after glucose injection.
Comments	

REFERENCES

Al-Zubaidi, A., Heldmann, M., Mertins, A., Jauch-Chara, K., and Münte, T. F. (2018). Influences of Hunger, Satiety and Oral Glucose on Functional Brain Connectivity: A Multimethod Resting-State fMRI Study. *Neuroscience* 382, 80–92. doi:10.1016/j.neuroscience.2018.04.029

Al-Zubaidi, A., Iglesias, S., Stephan, K. E., Buades-Rotger, M., Heldmann, M., Nolde, J. M., et al. (2020). Effects of hunger, satiety and oral glucose on effective connectivity between hypothalamus and insular cortex. *NeuroImage* 217, 116931. doi:10.1016/j.neuroimage.2020.116931

Flanagan, D. E., Fulford, J., Krishnan, B., Benattayallah, A., Watt, A., and Summers, I. R. (2012). Functional MRI of the hypothalamic response to an oral glucose load. *Diabetologia* 55, 2080–2082. doi:10.1007/s00125-012-2559-4

Liu, Y., Gao, J.-H., Liu, H.-L., and Fox, P. T. (2000). The temporal response of the brain after eating revealed by functional MRI. *Nature* 405, 1058–1062. doi:10.1038/35016590

Matsuda, M., Liu, Y., Mahankali, S., Pu, Y., Mahankali, A., Wang, J., et al. (1999). Altered hypothalamic function in response to glucose ingestion in obese humans. *Diabetes* 48, 1801–1806. doi:10.2337/diabetes.48.9.1801

Neudorfer, C., Germann, J., Elias, G. J. B., Gramer, R., Boutet, A., and Lozano, A. M. (2020). A high-resolution in vivo magnetic resonance imaging atlas of the human hypothalamic region. *Scientific Data* 7, 305. doi:10.1038/s41597-020-00644-6

Osada, T., Suzuki, R., Ogawa, A., Tanaka, M., Hori, M., Aoki, S., et al. (2017). Functional subdivisions of the hypothalamus using areal parcellation and their signal changes related to glucose metabolism. *NeuroImage* 162, 1–12. doi:10.1016/j.neuroimage.2017.08.056

Purnell, J. Q., Klopfenstein, B. A., Stevens, A. A., Havel, P. J., Adams, S. H., Dunn, T. N., et al. (2011). Brain functional magnetic resonance imaging response to glucose and fructose infusions in humans. *Diabetes, Obesity and Metabolism* 13, 229–234. doi:10.1111/j.1463-1326.2010.01340.x

Simon, J. J., Lang, P. M., Rommerskirchen, L., Bendszus, M., and Friederich, H.-C. (2023). Hypothalamic Reactivity and Connectivity following Intravenous Glucose Administration. *International Journal of Molecular Sciences* 24, 7370. doi:10.3390/ijms24087370

Simon, J. J., Stopyra, M. A., Mönning, E., Sailer, S., Lavandier, N., Kihm, L. P., et al. (2020). Neuroimaging of hypothalamic mechanisms related to glucose metabolism in anorexia nervosa and obesity. *The Journal of Clinical Investigation* 130, 4094–4103. doi:10.1172/JCI136782

Smeets, P. A. M., de Graaf, C., Stafleu, A., van Osch, M. J. P., and van der Grond, J. (2005a). Functional magnetic resonance imaging of human hypothalamic responses to sweet taste and calories. *The American Journal of Clinical Nutrition* 82, 1011–1016. doi:10.1093/ajcn/82.5.1011

Smeets, P. A. M., de Graaf, C., Stafleu, A., van Osch, M. J. P., and van der Grond, J. (2005b). Functional MRI of human hypothalamic responses following glucose ingestion. *NeuroImage* 24, 363–368. doi:10.1016/j.neuroimage.2004.07.073

Smeets, P. A. M., Vidarsdottir, S., de Graaf, C., Stafleu, A., van Osch, M. J. P., Viergever, M. A., et al. (2007). Oral glucose intake inhibits hypothalamic neuronal activity more effectively than glucose infusion. *American Journal of Physiology-Endocrinology and Metabolism* 293, E754–E758. doi:10.1152/ajpendo.00231.2007

Smith, S. M., Beckmann, C. F., Andersson, J., Auerbach, E. J., Bijsterbosch, J., Douaud, G., et al. (2013). Resting-state fMRI in the Human Connectome Project. *NeuroImage* 80, 144–168. doi:10.1016/j.neuroimage.2013.05.039

Teeuwisse, W. M., Widya, R. L., Paulides, M., Lamb, H. J., Smit, J. W. A., de Roos, A., et al. (2012). Short-Term Caloric Restriction Normalizes Hypothalamic Neuronal Responsiveness to Glucose Ingestion in Patients With Type 2 Diabetes. *Diabetes* 61, 3255–3259. doi:10.2337/db11-1835

van Opstal, A., Westerink, A., Teeuwisse, W., van der Geest, M., van Furth, E., and van der Grond, J. (2015). Hypothalamic BOLD response to glucose intake and hypothalamic volume are similar in anorexia nervosa and healthy control subjects. *Frontiers in Neuroscience* 9. doi:10.3389/fnins.2015.00159

Vidarsdottir, S., Smeets, P. A. M., Eichelsheim, D. L., van Osch, M. J. P., Viergever, M. A., Romijn, J. A., et al. (2007). Glucose Ingestion Fails to Inhibit Hypothalamic Neuronal Activity in Patients With Type 2 Diabetes. *Diabetes* 56, 2547–2550. doi:10.2337/db07-0193

Weaver, T. A. and Bucy, P. C. (1940). The anatomical relationships of the hypophysial stem and the median eminence. *Endocrinology* 27, 227–235. doi:10.1210/endo-27-2-227
